# Kinking Matters: *meta*-Terphenyl Improves Hydroxide Conductivity of Mechanically Robust Fluorine-Free Poly(arylene piperidinium) Copolymers for Anion Exchange Membranes

**DOI:** 10.1021/acsami.5c08476

**Published:** 2025-07-10

**Authors:** Kajari Mazumder, Hannes Nederstedt, Richard Weber, Shuichi Haraguchi, Richard Neubert, Felix A. Plamper, Christian Müller, Michael Sommer

**Affiliations:** † Institut für Chemie, 38869 TU Chemnitz, Professur Polymerchemie, Straße der Nationen 62, 09111 Chemnitz, Germany; § Department of Chemistry and Chemical Engineering, 11248 Chalmers University of Technology, 41296 Göteborg, Sweden; ∥ Institute of Physical Chemistry, 26545 TU Bergakademie Freiberg, Leipziger Straße 29, 09599 Freiberg, Germany; ⊥ Center for Efficient High Temperature Processes and Materials Conversion ZeHS, TU Bergakademie Freiberg, Winklerstr. 5, 09599 Freiberg, Germany; # Freiberg Center for Water Research ZeWaF, TU Bergakademie Freiberg, Winklerstr. 5, 09599 Freiberg, Germany; ∇ Forschungszentrum MAIN, TU Chemnitz, Rosenbergstraße 6, 09126 Chemnitz, Germany

**Keywords:** anion exchange membranes, poly(arylene piperidinium) copolymers, hydroxide conductivity, fluorine-free polymers, *meta*-terphenyl polymers, polymer electrolyte membranes

## Abstract

Poly­(arylene piperidinium) (PAP) polymers have emerged as promising candidates for applications as anion exchange membranes (AEMs) and have seen some commercial use in the form of PiperION by Versogen; however, PiperION contains fluorinated units to balance its ionic content. Fluorine-free variants are environmentally more friendly alternatives as recycling is facilitated. Herein, we report a series of four fluorine-free PAP membranes that are mechanically robust and feature moderate water uptake yet high ionic conductivity. *p-*Quaterphenyl (*p*QP) is copolymerized with either *m-* or *p-*terphenyl (*m/p*TP) and *N*-methyl-4-piperidone under superacid-catalyzed polyhydroxyalkylation conditions. The molar ratios of the reactants are adjusted to maintain a balance of solubility and flexibility of the polymers and to reach ion exchange capacities between 2.53 and 2.66 mequiv g^–1^. The polymers exhibit thermal stability of *T*_d,95_ > 260 °C, Young’s moduli between 0.7 and 1.0 GPa, and ultimate tensile stresses of 50–60 MPa in the dry state. Additionally, under submersion tensile deformation, the Young’s moduli and ultimate tensile stresses are in the range 200–320 MPa and 15–22 MPa, respectively. The sample with an equimolar ratio of *p*QP and *m*TP was found to exhibit a robust nature with elongation up to 170% when subjected to submersion tensile deformation, thus showing attractive mechanical properties under relevant working conditions. Wet membranes show an ionomer SAXS peak in the range of 5 nm, suggesting clustering of water and ionic parts of the chain. High hydroxide conductivity of up to 197 mS cm^–1^ at 80 °C is observed. Such behavior is promising considering their water uptake of 85% at 80 °C as an upper limit, resulting in moderate areal and through-plane swellings of 100% and 55%, respectively. The results demonstrate that fluorine-free PAPs can be tuned to match important criteria of AEMs, including low water uptake, high dimensional and alkaline stability, and high hydroxide conductivity.

## Graphical abstract

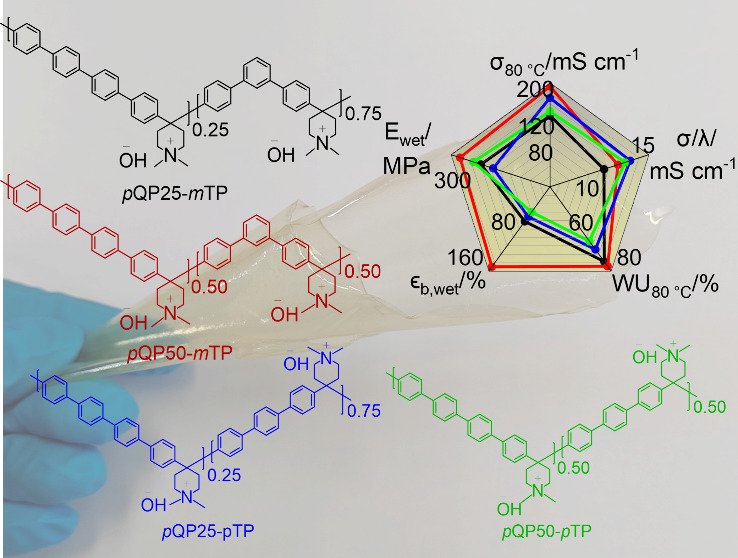

## 1 Introduction

Water electrolysis is a key technology in the production of green hydrogen, a clean fuel with potential to drive industries such as transportation, manufacturing, and energy storage to carbon-free alternatives.^1,2^ When driven by renewable energy sources such as wind or solar power, this technology produces hydrogen without emitting any greenhouse gases, which makes it appealing for sustainability initiatives. At present, the three main methods for water electrolysis are based on alkaline water electrolysis (AWE), proton exchange membrane water electrolysis (PEMWE), and anion exchange membrane water electrolysis (AEMWE).^3^ AWEs have the advantage of operating without platinum-group metal (PGM) catalysts; however, they work under high alkaline conditions (5 M KOH solution), inducing safety and operation issues and limited efficiency in producing pressurized hydrogen.^4^ PEMWEs are promising alternatives in terms of their higher efficiency and higher current density; however, their dependency on PGM catalysts for mass production of hydrogen is a driving force to search for other alternatives.^5^ The advent of Nafion brought about a breakthrough in the field of proton exchange membrane applications; however, Nafion and its derivatives suffer from high production costs and belong to the class of poly- and perfluorinated (PFAS) substances, which are toxic compounds that are potentially affected by legislative restrictions in the future. Despite a good number of fluorine-free PEM membranes with competitive properties being available,^6–8^ PFAS-based membranes continue to be widely used.^9–11^

In contrast, water electrolysis based on AEMs can combine the advantages of both PEMWE and AWE. AEMs in alkaline fuel cells or water electrolyzers can function with PGM-free catalysts based on Co, Ni, Fe, and Mn, and cell design is possible in the absence of corrosion-resistant materials such as titanium. Thus, AEMWEs prove to be better alternatives as they can lower the need for high KOH concentration as is required in AWEs and also produce hydrogen at higher pressure. AEMWEs can also overcome some of the cost issues related to PEMWEs, thereby promising a sustainable mass production of green hydrogen.^12,13^ However, despite the above advantages the commercialization of AEMs is limited as they suffer from limited durability and poor conductivities as compared to PEMs.^14,15^

It is also a challenge to have sufficiently stable membranes in high alkaline conditions. AEMs prepared by conventional nucleophilic polymerization reactions often end up having heteroatoms in the polymer backbone which are susceptible to degradation in high alkaline medium.^16^ All carbon-based polymer backbones can however be efficiently prepared using the Friedel–Crafts-type superacid-catalyzed polycondensation, and they are generally not susceptible to any degradation under alkaline conditions.^17,18^ Furthermore, the positively charged cationic groups responsible for the hydroxide ion transport in an AEM are prone to hydroxide attack primarily via Hofmann elimination and nucleophilic substitution reactions.^19^ Several quaternary ammonium (QA) groups have been extensively studied because of their availability and high alkaline content.^20–23^ Six-membered piperidinium-based cationic groups have been found to demonstrate the least reactivity in nucleophilic substitution and elimination reactions at high pH and high temperature.^24,25^

Another limiting factor for commercial applications of AEMs is their low ionic conductivity (σ), resulting from the poor migration rate of hydroxide ions. This can be somewhat compensated by increasing the ionic content of the polymer, expressed as the ion-exchange capacity (IEC). However, a higher ionic content of the polymer leads to higher water uptake (WU) and swelling ratio (SR), consequently lowering the mechanical strength of the polymer membrane.^26,27^ The synthesis of poly­(arylene piperidinium)­s has been extensively studied using a variety of arene monomers to precisely tailor the physicochemical properties of AEMs. To balance hydrophobicity and water uptake, the design and choice of the arene are of utmost priority. Notably, poly­(arylene piperidinium)­s synthesized from *m*-terphenyl exhibit markedly higher water uptake compared to their *p*-terphenyl-based counterparts. This difference arises from the increased backbone flexibility of *m*-terphenyl-based polymers, which facilitates greater water absorption and swelling.^28,29^ Some of the other common arenes reported in the literature are biphenyl,^30,31^ *m-*terphenyl,^32,33^ *p-*terphenyl,^34,35^ acenaphthylene,^36^ 9,9′-dimethylfluorene,^37,38^ 9,10-diphenylanthracene,^39^ 1,3,5-triphenylbenzene,^40^ and 1,2-dibenzylethane.^41^ In general, the incorporation of more rigid aromatic monomers leads to reduced water uptake and enhanced mechanical robustness due to restricted polymer chain mobility.^42^ Furthermore, homopolymers of terphenyl and piperidinium cations result in high water uptake (WU > 100% at room temperature).^43^ To minimize the WU, cross-linking^44^ or copolymerization of electron-rich arenes like biphenyl, *meta-*terphenyl, or *para-*terphenyl with a nonionic ketone monomer is an often adopted strategy.^30–35,45^

A study by Liu et al. demonstrated that poly­(arylene piperidinium) incorporating *p*-quaterphenyl (*p*QP) units exhibited improved phase separation, superior membrane-forming ability, and controlled water swelling behavior when compared to poly­(arylene piperidinium) based on shorter biphenyl and *p*-terphenyl units.^46^ The work described above also reported on the excellent solubility of the homopolymer from *p*QP and *N*-methyl-4-piperidone. However, our attempts indicated that copolymerization with at least 50 mol % *m-* or *p*-terphenyl (*m/p*TP) was necessary to ensure sufficiently soluble polymers with *p*QP in the backbone. Nevertheless, when compared to similar copolymers with terphenyl in the polymer backbone, polymers prepared in the current study exhibited lower water uptake, despite having higher IEC than those reported by Liu et al.^46^ These findings highlight the potential of statistical copolymers as well as the crucial role of monomer selection in optimizing the AEM properties. Bakvand et al. further modulated *meta*-kinks in a series of alternating quaterphenyl-piperidone copolymers and found that central membrane characteristics such as water uptake and hydroxide conductivity were strongly dependent on the connectivity of the arene monomer.^47^ However, these polymers showed large values of water uptake (WU ∼ 200% at 80 °C), which were too large for membranes with reasonable mechanical robustness.

Here we report on a series of synthetically simple, statistical poly­(arylene piperidinium) copolymers based on *p*QP and terphenyl, with backbones that are entirely ether- and fluorine-free. The polymers are referred to as *p*QPx-*m/p*TP, where x denotes the feed ratio of *p*QP (*p-*quaterphenyl) and *m/p* stands for either *meta*- (*m*TP) or *para*-terphenyl (*p*TP). By copolymerizing *N*-methyl-4-piperidone with different molar ratios of *p*QP and either *m*TP or *p*TP, we prepared four copolymers with comparable IEC values. Combining *p*QP with *m*TP furnishes soluble copolymers with high and comparable molar masses between *M̅*_n,SEC_ = 50 and 65 kg/mol, excellent mechanical properties in the wet state (*E*_wet,RT_ = 15–20 MPa, σ_wet,RT_ = 200–300 MPa, ε_b,wet_ = 60–170%), and high hydroxide conductivities of up to 197 mS/cm. The presence of *p*QP in the backbone increases chain rigidity and hydrophobicity, thereby limiting WU and swelling, and simultaneously improves mechanical strength. Shares of *p*TP and *m*TP improve the solubility and thus facilitate the synthesis of high molar mass materials as well as membrane casting. The *meta*-kinks introduced by *m*TP result in solution processable membranes, lead to larger strain at break values, and only slightly increase WU compared to *p*TP analogs. The copolymer with an equimolar mixture of *p*QP and *m*TP yields an optimized property profile featuring the best mechanical properties, the highest hydroxide conductivity at 80 °C, and a moderate WU. Additionally, the alkaline stability of the membranes was also studied under different alkaline conditions, resulting in stabilities that are at least comparable to similar reported membrane materials. For a better comparison of polymer properties, we have added data from a reference polymer termed M0 ^48^ which has a similar structure to PiperION-A.

## 2 Experimental Section

### 2.1 Materials

*p*-Quaterphenyl (*p*QP, Aldrich, 98%), *m-*terphenyl (*m*TP, BLD Pharm, 99.5%), *p-*terphenyl (*p*TP, Acros Organics, 99%), *N*-methyl-4-piperidone (BLD Pharm, 98.27%), dichloromethane (DCM), trifluoroacetic acid (ABCR, 99.9%), trifluoromethanesulfonic acid (ABCR, 99%), dimethyl sulfoxide (DMSO), potassium carbonate (K_2_CO_3_, Grüssing, 99.5%), and methyl iodide (MeI, Thermo Scientific, 99%) were used as received.

### 2.2 Synthesis of Poly­(*p*-quaterphenyl *N*-methyl-4-piperdinium-*co*-*m/p*-terphenyl *N*-methyl-4-piperdinium) Copolymers

Copolymerization of *p*-quaterphenyl and either *m-*terphenyl or *p*-terphenyl together with *N*-methyl-4-piperidone was based on a previously published procedure,^46^ and the polymerization using a molar ratio of 1:1 of *p*-quaterphenyl:*m*-terphenyl is given as an example. Amounts of 3.289 g (29.1 mmol) *N*-methyl-4-piperidone, 3.425 g (11.2 mmol) *p*QP, and 2.5744 g (11.2 mmol) *m*TP were added to a 250 mL 2 neck round-bottom flask equipped with a mechanical stirrer. Subsequently, 19.80 mL of DCM was added, and the mixture was cooled to 0 °C under stirring. At this temperature, 2.2 mL (29.1 mmol) of TFA and 20 mL (224 mmol) of TFSA were added. The mixture was stirred at 0 °C for 2 h and then allowed to reach room temperature. After an additional 6 h of stirring, the mixture was poured into an excess of aq. 1 M NaOH solution at 0 °C. The solid was cut into pieces and stirred in concentrated aq. NaHCO_3_ overnight. Subsequently, the solid was filtered and washed with water until the filtrate was neutral. The solids were washed with methanol and vacuum-dried at 60 °C, furnishing ∼7.9 g of a white fibrous solid.

### 2.3 Methylation of Copolymers

The obtained copolymers were directly methylated using a modified literature method.^46^ The methylation of a polymer with a molar ratio of *p*QP:*m*TP of 1:1 is given as an example. An amount of approximately 12 g of polymer was added to a round-bottom flask together with 126 mL of DMSO, 3.135 g (22.4 mmol) of K_2_CO_3_, and 4.18 mL (67.1 mmol) of MeI. The mixture was stirred for 1 day at room temperature under protection from light. Subsequently, the mixture was centrifuged and the obtained supernatant precipitated dropwise to a 4:1 (v:v) solvent mixture of diethyl ether and isopropanol. The solids were separated by filtration, washed with fresh solvent mixture, and collected in a beaker. The polymer was ion-exchanged to chloride form by stirring in an aqueous 1 M potassium chloride solution for 3 days, followed by filtration and washing with water until no chloride ions were detected in the filtrate by using an aqueous 0.05 M AgNO_3_ indicator. The solid was dried under vacuum at 60 °C overnight yielding 7.519 g (81%) of *p*QP50-*m*TP. For ^1^H NMR spectra see Figure S1.

*p*QP25-*m*TP: ^1^H NMR (DMSO-*d*_6_): δ (ppm) 7.55 (d; *D*), 3.38 (s; *B*), 3.12 (s; *A*), 2.84 (s; *C*).

*p*QP50-*m*TP: ^1^H NMR (DMSO-*d*_6_): δ (ppm) 7.64 (d; *D*), 3.39 (s; *B*), 3.13 (s; *A*), 2.85 (s; *C*).

The copolymers containing *p*TP in their backbone were prepared using the same method. For ^1^H NMR spectra see Figure S2.

*p*QP25-*p*TP: ^1^H NMR (DMSO-*d*_6_): δ (ppm) 7.62 (d; *D*), 3.37 (s; *B*), 3.11 (s; *A*), 2.86 (s; *C*).

*p*QP50-*p*TP: ^1^H NMR (DMSO-*d*_6_): δ (ppm) 7.60 (d; *D*), 3.39 (s; *B*), 3.13 (s; *A*), 2.85 (s; *C*).

### 2.4 Membrane Casting

The quaternized polymers were dissolved in dimethyl sulfoxide at a concentration of ∼5 wt%. These solutions were subsequently filtered through 0.45 μm Teflon filters, and the solvent was removed under a vacuum at 60 °C overnight. Then, the polymers were dissolved in DMSO again at a concentration of 20–30 wt%. The concentrated solutions were spread over a glass substrate at room temperature using a doctor blade, and the solvent was removed at ambient pressure at 60–80 °C during 24 h. The resulting membrane was removed by soaking the whole membrane in deionized water at 60 °C followed by peeling the membrane off the substrate. Subsequently, the membrane was extensively washed in deionized water at 60 °C to remove any remaining solvent.

### 2.5 Polymer Characterizations

#### NMR Spectroscopy

^1^H NMR spectra were recorded in DMSO-*d*_6_ (δ ^1^H = 2.50 ppm) by using an Avance NEO 600 FT spectrometer (600 MHz). Solutions contained 3 mol % trifluoroacetic acid to protonate any tertiary amines as well as caused a downfield shift of the water signal.

#### Size Exclusion Chromatography (SEC)

Molecular weights were measured on a Shimadzu system comprising a 10 μm PSS GRAM guard column and three PSS GRAM columns with pore sizes ranging from 30 to 10^3^ Å, connected in series with an RID20A refractive index detector and an SPD-40 V UV–vis detector (Shimadzu). Calibration was done with polystyrene standards. A 0.1 M solution of ammonium trifluoroacetate in DMF was used as an eluent at 70 °C with a flow rate of 1.0 mL min^–1^.

#### Thermal Gravimetric Analysis (TGA)

TGA was measured on membrane samples in chloride form under a nitrogen atmosphere using a Thermogravimetric Analyzer from PerkinElmer Company. Samples were heated to 120 °C and kept isothermally at this temperature during 30 min, after which they were cooled to 30 °C. Lastly, the samples were heated to 650 °C with a heating rate of 10 °C min^–1^.

#### Tensile Testing

Stress–strain experiments for the membranes in chloride form were carried out under ambient conditions using a Linkam TST-350 with a displacement ramp of 5 μm s^–1^. These measurements were carried out on dog bones of membranes prepared using a cutting die in which the gauge was 15 mm × 2 mm. Ultimate tensile strength (UTS) and elongation at break (*ε*_b_) were taken as the stress and strain, respectively, at the point of sample fracture. Young’s moduli (E) were obtained by linear regression of the initial linear part in the stress–strain curves.

Mechanical properties in the wet conditions were measured on an Instron 68TM-5 Universal Testing System (Instron, Norwood, MA, US) equipped with submersible grips and a temperature-controlled BioBath. 20 mm × 2 mm rectangular specimens were used, which were deformed until failure at a crosshead speed of 10 mm min^–1^. The specimens were kept in deionized water for more than 5 days until just before the measurement at room temperature. Before each measurement, the specimen was removed from the deionized water bath and immediately clamped in the Biobath, which was filled with deionized water and equilibrated for about 5 min before starting the tensile test. To obtain the average mechanical properties for every membrane material, a minimum of three samples were examined under the same conditions.

#### Small-Angle X-ray Scattering (SAXS)

SAXS experiments of the *p*QPx-*m/p*TP copolymer membranes in hydroxide form were performed at a SAXSpoint 5.0 beamline (Anton Paar, Graz, Austria, measurement software SAXSdrive Ver. 3.04.1.1055) equipped with a Primux 100 microfocus X-ray source (Cu Kα radiation; λ = 1.54Å), ASTIX 2D multilayer X-ray optics, and a 2D EIGER2 R 1 M hybrid photon counting detector shielded with a mylar film (Dectris, Baden, Switzerland). The beam diameter was about 0.2 mm, and the sample detector distance was 42 cm. The measurement duration was chosen as 8.3 h per sample. Prior to analysis, the membranes (30 mm × 10 mm) were stored in degassed DI water at RT. The analysis was performed under ambient conditions (20 °C) using a sample holder with Kapton (Dupont) windows. The signal arising from this material was masked in the data (3.3 ≤ *q* [nm^–1^] ≤ 5.5). The data processing was performed with SAXS analysis (Anton Paar, Graz, v. 4.50.0.470), and the detailed description is as follows. A zero-point calibration was performed by using the primary beam position. All device-dependent influences, such as the beam stop, were masked. The data was transformed in *q* space and radially averaged to obtain the 1D curve. After a logarithmic binning from 0.1 to 7 nm^–1^ in 1000 bins, all data were normalized by the maximum intensity of the Kapton signal between 3 and 5.5 nm^–1^. Afterward, the background of pure water was subtracted; the Kapton range was masked; and the data was binned again logarithmically from 0.6 to 6 nm^–1^. The data was analyzed by fitting a Gaussian peak in the range from 1.3 ≤ *q* [nm^–1^] ≤ 3.3 using SASview (Vers. 6.0.0) (eq 1):

1 $$I\left(q\right)=\left(\mathrm{scale}\right)\cdot e^{-{\left(q-q_{0}\right)}^{2}/2\sigma^{2}}+\mathrm{background}$$

The fitting parameters for Gaussian fits of scattering data are given in Table S1. The characteristic separation distance in the membranes was calculated using Bragg’s equation as given in Equation S1.

#### Brunauer–Emmett–Teller (BET) Gas Sorption Measurements

The nitrogen adsorption–desorption isotherms were recorded at 77 K using an Autosorb IQ2 analyzer from 3P Instruments on membrane samples. Specific surface areas were calculated by BET gas sorption measurements, and the pore size distributions were calculated from the adsorption isotherm using the quenched solid density functional theory method, assuming slit-type pores.

#### Water Uptake (WU) and Swelling Ratio (SR)

The water uptake of the membranes was measured in the hydroxide form according to the following method. Membrane pieces in chloride form were ion-exchanged to hydroxide form by immersion in carbon dioxide-free 1 M sodium hydroxide solution for 3 days at room temperature. Then, the membranes were first extensively washed with and stored in degassed deionized water. After 2 days of storage at room temperature, the membranes were removed, gently wiped with tissue paper, and weighed. This procedure was repeated after 2 days of storage at 40, 60, and 80 °C. Subsequently, the membranes were ion-exchanged to chloride form by immersion into an aqueous 1 M KCl solution during 2 days at 60 °C, after which they were extensively washed with deionized water and dried under vacuum at 60 °C during 1 day. The dry membranes in the chloride form were weighed, and their weight in the hydroxide form was calculated by multiplying the noted weights with the ratio of the molar mass of the repeating unit in the hydroxide form and the molar mass in the chloride form. The water uptake was calculated as the mass increase of the wet membranes divided by the dry weight. From these values, the hydration number, defined as the molar concentration of water divided by the concentration of hydroxide ions, was calculated by dividing the water uptake by the product of the theoretical ion-exchange capacity (IEC, in hydroxide form) × the molar mass of water.

#### Alkaline Stability

Copolymer membranes in hydroxide form were immersed into 5 M KOH for 7, 14, 21, and 28 days at 80 °C. To obtain additional stability data for comparison with the literature, membranes in hydroxide form were also immersed into 2 M NaOH for 30 days at 90 °C. ^1^H NMR spectroscopy was used to evaluate the extent of the degradation. The membranes were first taken out from the KOH solution and thoroughly washed with DI water. Then they were ion exchanged to chloride form using 1 M KCl solution, and afterward they were dried for 48 h under vacuum at 60 °C.

#### Ion Conductivity (σ)

The membranes with dimensions 4 × 0.5 cm^2^ in chloride form were immersed in 1 M NaOH for 2 days. The membranes were then immersed in deionized water for 1 day at rt, and their thickness was determined. To remove carbonate ions unintentionally produced from the reaction with CO_2_, a current of 0.1 mA was applied to the source electrodes. After attaining constant resistance values over a period of 7 h, the membrane resistance (*R*) was measured at 25, 40, 60, and 80 °C by electrochemical impedance spectroscopy (EIS) over the frequency range of 50 to 7 × 10^6^ Hz. The ion conductivity was determined using eq 2 as follows:

2 $$\sigma =L\cdot {\left(R\cdot W\cdot T\right)}^{-1}$$

where *L* is the distance between the electrodes (set at 0.425 cm), *W* is the width of the membranes, and *T* is the membrane thickness after immersion in deionized water at room temperature.

#### Ion Exchange Capacity

The IEC values of the membranes in chloride form were determined using a TitroLine 7000 (SI Analytics) with a AgCl 62 electrode. The membranes were dried at 60 °C in a vacuum oven for 2 days and cut into pieces of 3 × 3 cm^2^. Then they were weighed (*w*_dry_) and immersed into a 1 mol L^–1^ Na_2_SO_4_ solution for 3 days under stirring to replace chloride by sulfate.^49,50^ The solution was titrated using a 0.01 mol L^–1^ AgNO_3_ solution, and the equivalence point was determined with a slope value of 400 mV/mL. The IEC of membrane samples was obtained using eq 3 as follows:

3 $$IEC=\frac{c_{{AgNO}_{3}}\times V_{{AgNO}_{3}}}{w_{dry}}$$

where *c*_AgNO_3__ (mol L^–1^) and *V*_AgNO_3__(L) denote AgNO_3_ concentration and volume at the equivalence point, respectively.

## 3 Results and Discussion

### 3.1 Synthesis and Molecular Characterization

The four copolymers were prepared by superacid-mediated polyhydroxyalkylation. Sufficiently soluble poly­(*p*-quaterphenyl piperdinium) homopolymers were not obtained in our hands following the procedure of Liu et al.,^46^ despite further optimization of these procedures. Instead, we observed that the addition of at least 50 mol% *m*TP or *p*TP was required to obtain sufficiently soluble polymers. The chemical structures of the synthesized polymers are listed in Figure 1. The polyhydroxyalkylations were performed by dispersing all monomers in dichloromethane (DCM), followed by the addition of trifluoroacetic acid and trifluoromethanesulfonic acid at 0 °C. The reaction mixture of *m*TP and *p*QP was dark purple in the beginning but turned to green after 2 h when gradually allowed to warm to room temperature. Simultaneously, the presence of nondissolved powder in the mixture drastically decreased. After 8 h, the dark green homogeneous mixture was poured into a sodium hydroxide solution at 0 °C yielding a fibrous solid that was cut into small pieces and washed with copious amounts of a concentrated, aqueous sodium bicarbonate solution followed by DI water and methanol. The solids were dried under vacuum at 60 °C. The polymers were quaternized using methyl iodide in dimethyl sulfoxide.^48^ After 1 day of reaction, the quaternized polymers were precipitated in a mixture of isopropanol and diethyl ether followed by ion exchange to chloride form and drying under vacuum at 60 °C. The obtained poly­(arylene piperidinium) copolymers were characterized by ^1^H NMR spectroscopy, indicating monomer conversion according to their feed ratios (Figures S1 and S2). ^1^H NMR spectroscopy confirmed that full quaternization was achieved as indicated by the expected integral ratios and the absence of any signals from protonated amines upon the addition of small amounts of TFA to the NMR solution. Polyhydroxyalkylations with *p*-terphenyl as a comonomer were performed in the same way as described. These reaction mixtures exhibited a dark blue color throughout the entire polymerization with less solid material in the mixture as the polymerizations proceeded.

**Figure 1:**
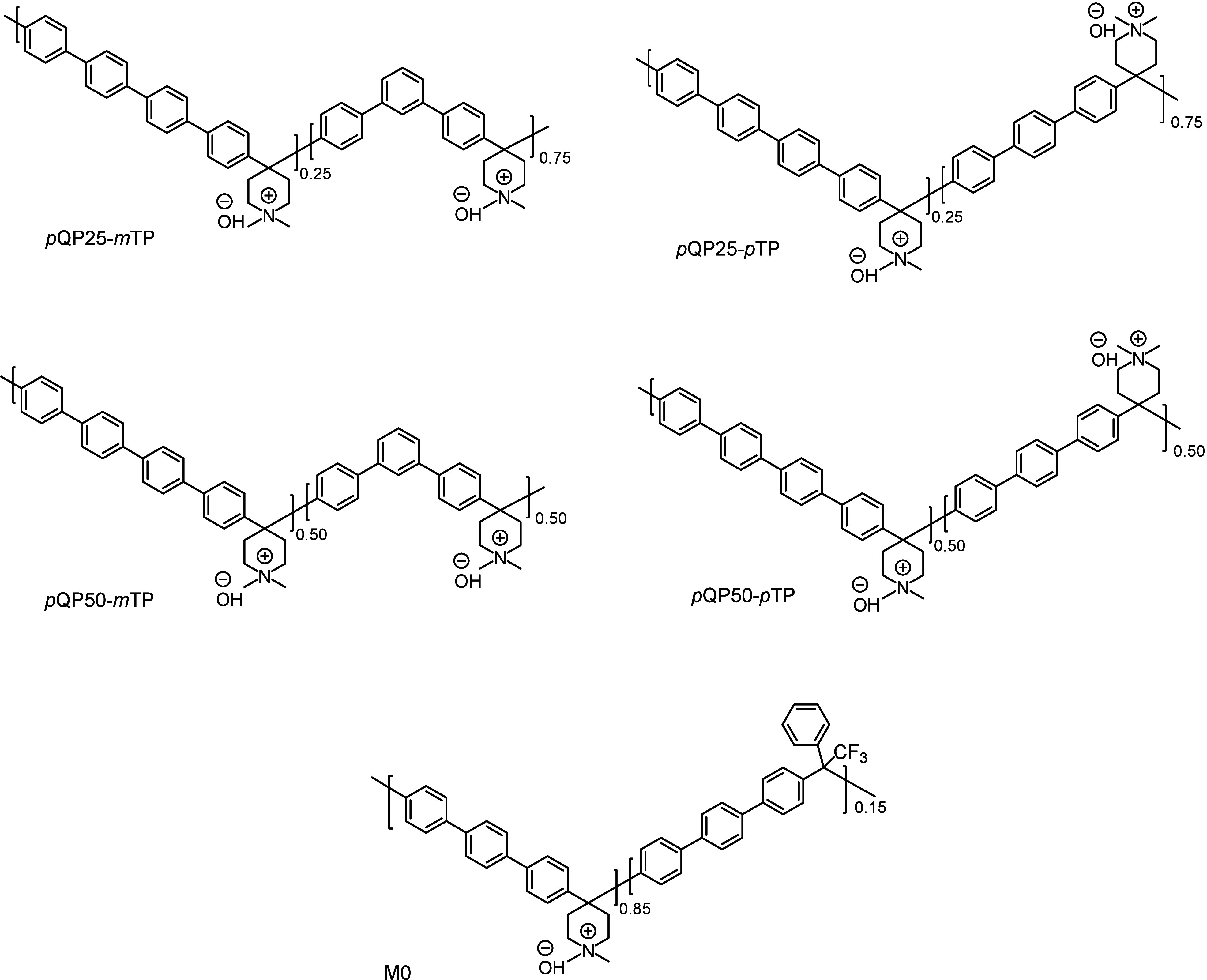
Chemical structures of the hydroxide forms of *p*QPx-*m/p*TP copolymers. M0 is a reference polymer with a structure similar to PiperION-A.

The molar mass distributions were obtained by size-exclusion chromatography (Figure S3) and calibrated against polystyrene standards. The number-average molar mass (*M̅*_n_) and dispersity (*Đ*) values are reported in Table 1. The *M̅*_n_ values of the quaternized polymers were between 53 and 65 kg mol^–1^, which is typically sufficient for membrane forming properties, and the value of the molar mass dispersity was around 2. An image of *p*QP50-*m*TP is supplied in Figure S4. The IEC values of the *p*QPx-*m/p*TP copolymers in Cl^–^ were determined from the ^1^H NMR spectra as well as potentiometric titrations and are listed in Table 1. The IEC values of the polymers were found to be between 2.41 and 2.53, which agree with the respective values from potentiometric titrations.

**Table 1:** Molecular Properties, Surface Area, Pore Size, Thermal Properties, and Ion Exchange Capacity of *p*QP*x*-*m/p*TP Copolymers and M0

				BET analysis^b^			IEC^c^/meq g^–1^	
copolymer	feed ratio *p*QP/*mX/p*TP	*M̅*_n_^a^/kg mol^–1^	*Đ* ^a^	Surface area/m^2^ g^–1^	pore size/Å	*T*_d,95_/°C	theoretical	titration
M0	0/100	49	2.1	15.5	/	224	2.19	2.14 ± 0.10
*p*QP25-*m*TP	25/75	53	2.1	13.1	210	282	2.53	2.45 ± 0.20
*p*QP50-*m*TP	50/50	65	2.1	2.5	200	278	2.41	2.33 ± 0.15
*p*QP25-*p*TP	25/75	56	2.2	16.9	206	259	2.53	2.48 ± 0.32
*p*QP50-*p*TP	50/50	53	2.0	3.0	406	255	2.41	2.28 ± 0.20

a Number-average molar mass and dispersity determined in trifluoracetate form by SEC in DMF with 0.1 M ammonium trifluoroacetate.

b Using membranes.

c Ion exchange capacity of copolymers in chloride form.

### 3.2 Thermal Stabilities

To assess the thermal stability of the polymers, thermogravimetric analysis (TGA) was performed under a nitrogen atmosphere (Figure 2, Table 1). To distinguish pure thermal decomposition from the effects of alkaline stability, the measurements were performed on membranes in chloride form. The temperature at 5% weight loss (*T*_d,95_) was used to quantify the thermal stability. All polymers showed *T*_d,95_ between 255 and 285 °C, which is suitable for AEMWE operation. When comparing the samples containing *m*TP and *p*TP, it was found that the *T*_d,95_ of copolymers containing *p*TP was lower by approximately 20 °C than those containing *m*TP. Upon comparing the two polymers with different shares of *m*TP, slightly larger *T*_d,95_ values were found for *p*QP25-*m*TP with higher *m*-terphenyl content (278 °C versus 285 °C). These results are consistent with a study from Long et al., who observed an earlier decomposition of poly­(*p*-terphenylene *N*,*N-*dimethylpiperidinium) compared to poly­(*m*-terphenylene *N*,*N-*dimethylpiperidinium).^28^ This may indicate that *p*-arylene segments, including also the herein used *p*QP, have slightly lower thermal stability, possibly as a result of a less dense packing. However, this incorporation simultaneously leads to a higher weight fraction of more stable arylene content, thereby leading to similar decomposition behavior for the two copolymers with *m*TP, while the decomposition of the more rigid copolymers with *p*TP increased with increasing share of *p*QP. Upon further heating, all four polymer samples exhibited three distinct decomposition steps at approximately 310, 415, and 575 °C, respectively, as seen by the inflection points in Figure 2a and local maxima in the (negative) temperature derivative in Figure 2b. In particular, the second step showed the greatest variance between the four samples with values ranging from 408 to 432 °C. Previously, the first and second decomposition steps were assigned to decomposition of the piperidinium and arylene units, respectively.^28,51^ In agreement with the stiffness induced by the *p*-arylene backbones, during these steps also the copolymers with *p*TP polymers exhibited a higher degree of decomposition than copolymers with *m*TP. Although the polymers reported in this work show higher degradation temperature than M0, a significant difference in the nature of thermograms with respect to molar ratio of *p*QP and/or *m-*/*p*TP was not observed.

**Figure 2:**
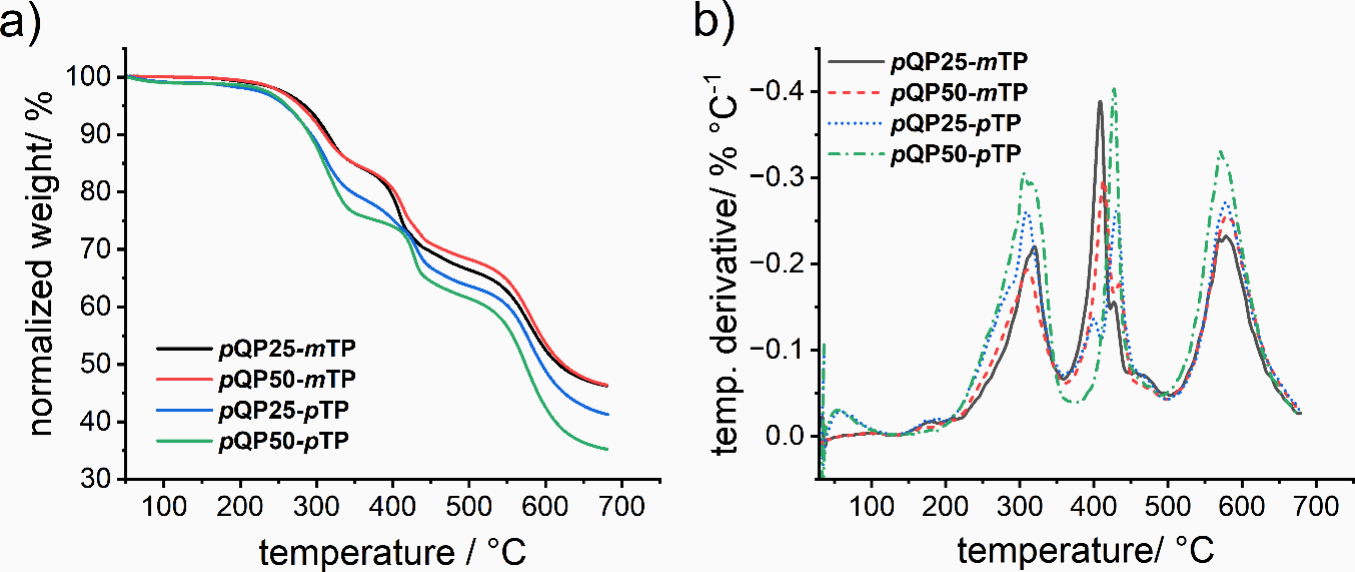
a) TGA thermograms showing remaining weight as a function of temperature and b) corresponding temperature derivative. The latter was calculated by numerical differentiation followed by smoothing using a moving local regression using weighted linear least-squares and a 2nd degree polynomial model in MATLAB.

### 3.3 Mechanical Properties

Tensile deformation in low as well as highly humid environments was used to assess the mechanical strength and flexibility of the membrane when encountered in electrolyzers. In the present study, the tensile deformation measurements were performed on dry membranes in chloride form under ambient conditions (Figure 3a) and also on immersed membranes in the hydroxide form (Figure 3b). The corresponding mechanical properties are given in Table 2.

**Figure 3:**
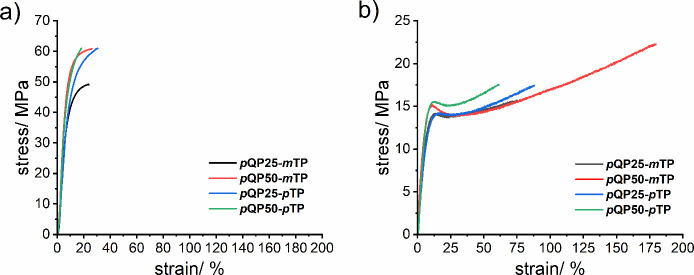
Stress–strain tensile deformation curves of *p*QPx-*m/p*TP in a) dry Cl^–^ forms under ambient conditions and in b) OH^–^ forms under submersed conditions.

**Table 2:** Mechanical Properties of Membranes in the Dry State under Ambient Conditions and under Submersed Conditions

	tensile test on dry membranes in Cl^**–**^ form^a^ ^,^ ^c^						submersion tensile test on membranes in OH^**–**^ form^b^ ^,^ ^c^					
	modulus/MPa		ultimate tensile strength/MPa		elongation at break/%		modulus/MPa		ultimate tensile strength/MPa		elongation at break	
copolymer	*E*	(max – min)/2	UTS	(max – min)/2	ϵ_b_	(max – min)/ 2	*E*	(max – min)/ 2	UTS	(max – min)/ 2	ϵ_b_	(max – min)/ 2
M0^d^	1491	493	65	2	31	6	128	16	13	4	47	36
*p*QP25-*m*TP	775	47	50	1	29	7	244	42	15	2	74	12
*p*QP50-*m*TP	1063	71	62	1	30	2	318	14	22	1	171	10
*p*QP25-*p*TP	754	141	61	7	30	1	201	24	16	2	67	18
*p*QP50-*p*TP	905	97	64	9	24	5	271	57	15	2	54	3

a Tensile test on dry membranes in Cl^–^ form in ambient conditions.

b Tensile test on membranes in OH^–^ form when immersed in water at 22 °C.

c Values represent the mean and max – min error of three to five measurements.

d Standard deviation values provided for M0 samples instead of max – min error.

Figure 4 shows a comparison of Young’s moduli (E), ultimate tensile strengths (UTS), and elongation at break (*ϵ*_b_) of the prepared *p*QPx-*m/p*TP copolymers in dry and submersed conditions. The Young’s moduli increase with increasing amount of *p*QP. The copolymers with *m*TP exhibited slightly higher values compared to analogs with *p*TP, for both dry and wet membranes. Similarly, values of UTS increase with increasing content of *p*QP. This is particularly visible for the two membranes containing *m*TP. Moreover, with a higher content of *m*TP, the membrane shows mechanical features comparable to that of M0 under dry conditions. With regard to elongation at break, clear trends are not observed upon variation of *p*QP, *m*TP, or *p*TP contents. These results may be compared to dry membranes prepared from poly­(*p*-quaterphenylene *N*,*N-*dimethylpiperidinium) by Gao et al. (albeit in hydroxide form under ambient conditions) that exhibited a similar UTS of 60 MPa but a lower elongation at break of 18%.^52^ From this comparison, it may be deduced that the herein used additional shares of *m*TP or *p*TP do not lead to significant changes in the UTSs but significantly increase elongation at break. A membrane prepared from a similar homopolymer was characterized mechanically in hydroxide form by Liu et al. at 30 °C at a relative humidity of 30%, yielding a UTS of 84 MPa, an *E* modulus of 2130 MPa, and an ϵ_b_ of 16%.^46^ While the two former values are larger than any reported for the membranes in this study, the values of elongation at break are smaller. This may be explained by the larger content of stiff *p*QP in the homopolymer, which generally increases mechanical strength but lowers elongation at break. Apart from these trends, a more detailed comparison of reported mechanical characterization and the herein shown data is not possible due to the different measurement conditions related to counterions and relative humidity.

**Figure 4:**
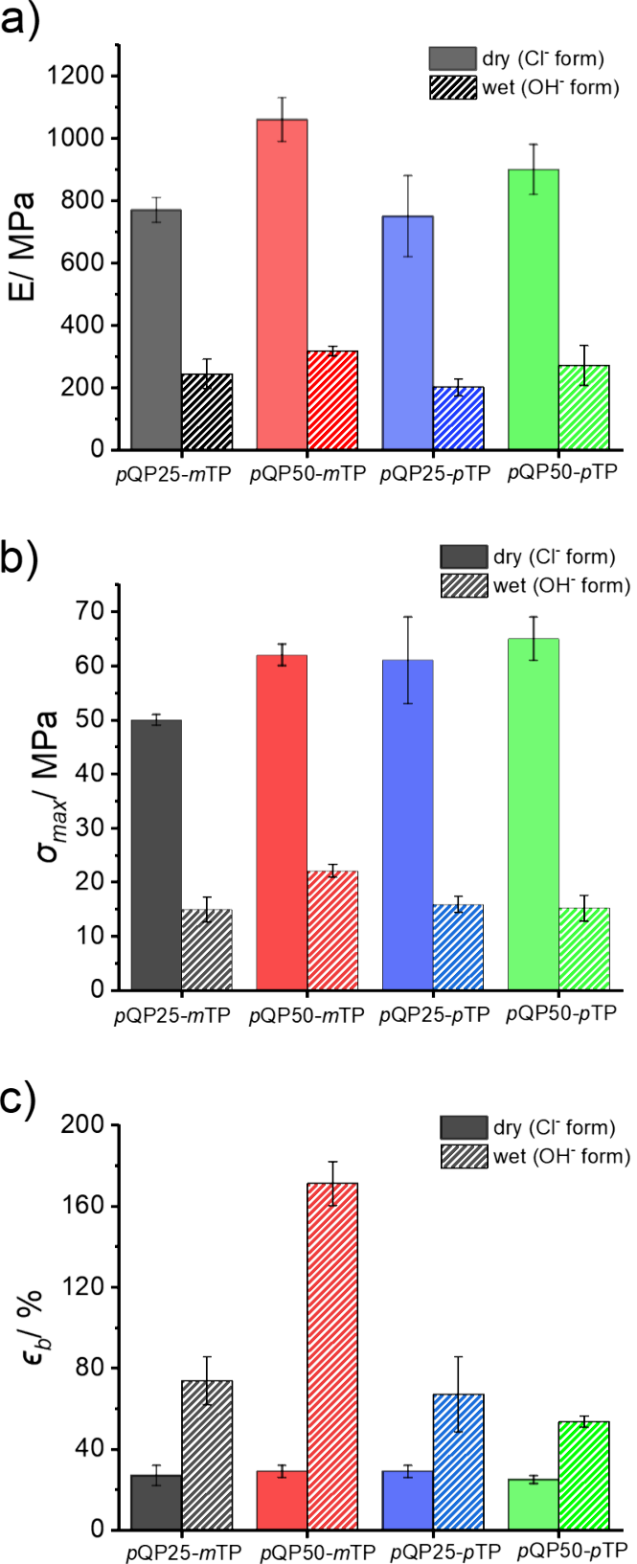
a) Young’s modulus (E), b) ultimate tensile strength (UTS) and c) elongation at break (*ϵ*_b_) of the dry chloride and fully hydrated hydroxide forms of *p*QPx-*m/p*TP. Values are given together with their min – max errors.

Additionally, the mechanical properties of fully hydrated membranes (OH^–^ forms) were determined by using tensile deformation under immersed conditions at room temperature. In general, at low humidity conditions, the quaternary ammonium ions show strong electrostatic interactions resulting in an increase in the stress and modulus of membranes.^53^ At highly humid conditions, the water molecules neighboring the quaternary ammonium groups give rise to a plasticizing effect, resulting in a high elongation at break but reduced stress and modulus.^54,55^ While for the fully hydrated membranes the tensile strength and modulus decreased, the elongation at break increased compared with dry membranes. Among the hydrated membranes, the *p*QP50-*m*TP membrane stands out by exhibiting the highest modulus, the highest ultimate tensile strength, as well as the highest elongation at break. This result is intriguing as both strength and elongation at break are improved and demonstrate the advantage of simple copolymerization to balance and optimize properties.

### 3.4 Membrane Morphology

To study the morphology of the membranes, small-angle X-ray scattering (SAXS) was performed on fully hydrated hydroxide forms of *p*QPx-*m/p*TP (Figure 5). We focused on periodic spacings due to the ionic clustering, which are expected in the *q*-range of ∼1 to 3 nm^–1^ for dry samples.^31,47,56^ SAXS of the pristine samples is not able to probe domain sizes beyond those seen in Figure 5. SAXS scans down to the limits of the device (0.03 nm^–1^) as shown in Figure S5, but within this range, no valuable signals could be observed. All membranes showed ionomer peaks due to clustering of ionic parts of the chain and water, with *q*-values between 1.2 and 1.5 nm^–1^. The corresponding domain spacings remained relatively similar across different samples, varying between 4.3 and 5.2 nm, where *p*QP50-*m*TP showed the smallest spacing and *p*QP25-*m*TP showed the largest spacing. Considering the typical water contents of the swollen films (30–40% at rt; see next section), hydrophilic domains are likely the minority component embedded in a hydrophobic matrix. Taking the typical distance between charges along the backbone, which is in the range of 1.5–2 nm, we anticipate that at least two hydrophobic ter-/quaterphenyl groups are required to bridge the hydrophilic domains, being approximately 5 nm apart. This observation could also indicate that not all ammonium groups are directly embedded in these hydrophilic domains but could act as sites for transport between the water-rich domains, which form probably a percolated (channel-like) network.

**Figure 5:**
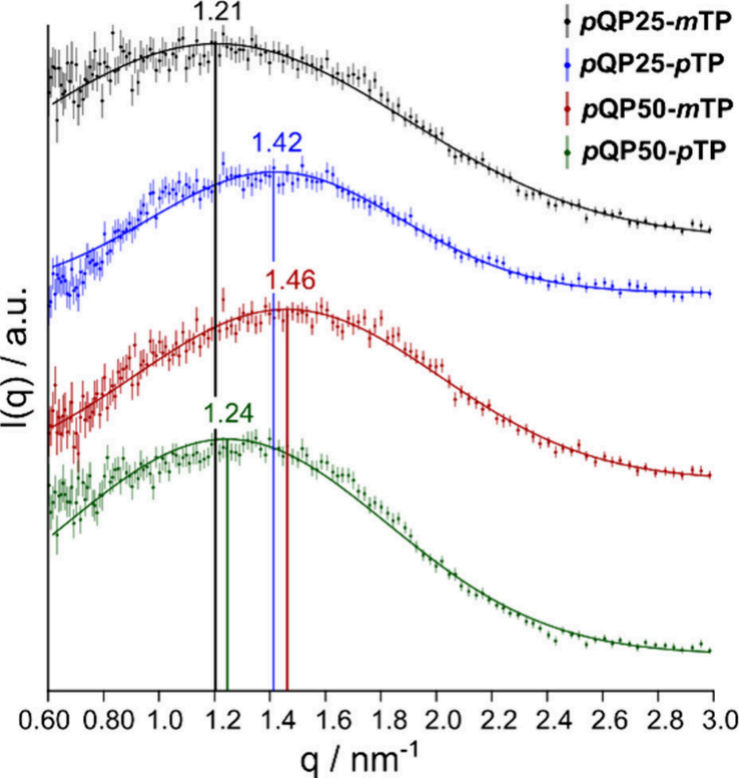
Small-angle X-ray scattering patterns (*q* range from 0.6 to 3 nm^–1^, *I*(*q*) – axis in linear scale) of *p*QPx-*m/p*TP copolymers and with Gaussian fits (for parameters see the SI). Membranes were measured in the hydroxide form and in the equilibrated wet state. Offset between the data sets established by equal amounts each (of arbitrary units) starting with the nonoffset normalized data of *p*QP50-*p*TP.

Generally, the ionomer peak arises from the electron density contrast between hydrophilic ionic domains (containing quaternary ammonium groups, counterions, and water) and hydrophobic polymer backbone regions in anion exchange membranes.^31,56^ This peak is a characteristic signature of microphase separation, representing the (near-range) regular spacing between ionic clusters that form a network of ion-conducting channels within the membrane matrix. As demonstrated by Pan et al.,^27^ these ionic channels are critical for hydroxide transport, where larger and more interconnected channels generally lead to improved ion mobility. The position of the peak (*q*_max_) is inversely proportional to the average separation distance between neighboring ionic domains (*d* = 2π/*q*_max_).

The domain spacing shows an inverse relationship with ion exchange capacity (IEC) values, as *p*QP50-*m*TP with the highest IEC exhibits the smallest spacing (4.3 nm), while *p*QP25-*m*TP with the lowest IEC shows the largest spacing (5.2 nm). This trend aligns with previous findings by Allushi et al.^56^ where higher ion concentrations lead to more compact ionic domains. The narrower spacing in high-IEC membranes suggests more densely packed ionic channels, likely contributing to the enhanced ionic conductivity observed in *p*QP50-*m*TP compared to *p*QP25-*m*TP. Liu et al.^46^ similarly found that well-defined ionomer peaks in quaterphenyl-based membranes corresponded to superior phase separation and ion transport properties.

Reproducibility measurements further revealed evidence for time-dependent morphological changes on longer time scales of up to 25 days, likely associated with CO_2_ absorption from the surrounding atmosphere (see SI, Figure S6). A complete understanding of these processes is beyond the scope of this manuscript.

### 3.5 Water Uptake, Hydration Number, and Swelling Ratio

The water uptake (WU) and corresponding hydration number (λ) and the swelling ratio (SR) of the membranes were measured according to the details given in section 2.5. The results of WU and λ are shown in Figure 6a and 6b, respectively. As expected, both WU and SR increase with increasing temperature, but the two copolymers containing *m*TP units swell stronger with temperature than the *p*TP analogs (Figure 6a).^48^ Reported homopolymers of type poly­(*p*-quaterphenylene *N*,*N-*dimethylpiperidinium) exhibited values of WU between 10 and 30% at temperatures of 20–80 °C;^46,52^ however, poly­(*p*-terphenylene piperdinium)­s have been studied in several instances with WU values of hydroxide forms ranging between 25 and 145% at 20 °C and between 40% and 400% at 80 °C.^28,40,43,57^ This broad range of WU values stems at least partially from differences in the molar masses of the polymers. Furthermore, there is no clear standard method of how to measure WU; for example, effects of equilibration time, carbon dioxide poisoning, and initial state or weight of membranes may all play a role. Here, the membranes were initially ion-exchanged to chloride form and then dried since drying of hydroxide forms may lead to degradation, loss of ionic groups, and, consequently, a decrease in water uptake. In any way, because of the differences in the details of the characterization methods, rendering a direct comparison with the literature is challenging. Membrane swelling, expressed in terms of areal and vertical swelling, is presented in Figures 6c and 6d. Overall, the trends of swelling follow those of the WU.

**Figure 6:**
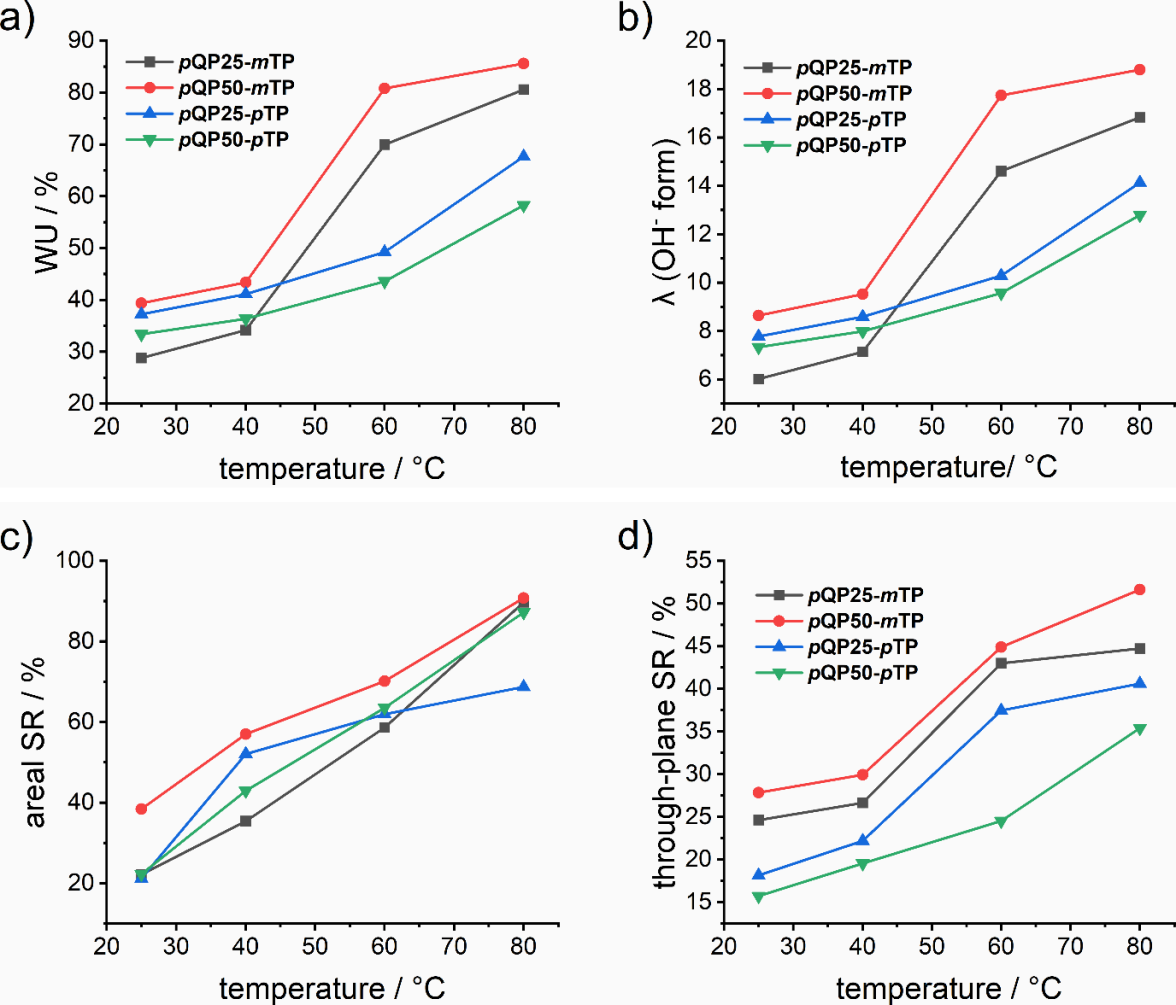
a) Water uptake, b) hydration number, c) areal swelling ratio, and d) through-plane swelling ratio of the membranes in hydroxide form as a function of temperature.

### 3.6 Hydroxide Conductivity

The hydroxide conductivity of the membranes was determined using two electrode impedance spectroscopy in deionized water at variable temperatures.^58^ As seen from Figure 7a, the conductivity of the prepared membranes in the hydroxide form (σ) increases with an increasing temperature as a result of enhanced diffusivity and water absorption. The membranes prepared in this work show higher conductivity as compared to previously reported AEMs based on *m*TP,^32,49^ with *p*QP50-*m*TP showing the highest conductivity of ∼197 mS cm^–1^ among the series. We attribute this result to the highest water uptake among the other polymers studied in this series and also to the less rigid *m*TP kinks that facilitate phase separation. The membrane *p*QP25-*m*TP has the highest content of *m*TP (75 mol %) but shows the lowest conductivity. This furthermore hints at how different feed ratios of *meta-* and *para-*arylenes may induce different pore sizes and distributions, and a high pore size may not always be optimum for high ionic conductivity.^44^ A morphology that is good for conductivity requires more interconnected voids and well-ordered ion channels. The surface area and pore sizes for the membranes as obtained from the BET analysis are listed in Table 1. Considering the low *d*-spacing from the SAXS measurements and a possible formation of thinner domains, hydroxide transport through a limited amount of water could be facilitated by enhanced percolation. The observation of the lowest *d*-spacings for samples with the highest water uptakes appears counterintuitive but could indicate an enhanced phase separation, especially where the kinked groups can arrange better around the water domains. For the nonkinked samples, the water might be even more spread out, leading in sum to slightly higher *d*-spacings. Furthermore, the apparent activation energy of the membranes was determined using the Arrhenius equation and was found to be in a similar range of 12–14 kJ mol^–1^ (Figure S7). Figure 7b shows the hydroxide conductivity vs hydration number plots for the synthesized polymers. Often, a high IEC and WU increase ionic conductivity; however, too large WU and SR can cause ion dilution and moreover weaken the membrane mechanically. Among the as-prepared membranes, *p*QP50-*m*TP shows the highest ionic conductivity (σ ∼ 197 mS cm^–1^ at 80 °C), which is significantly higher than that of M0 (σ = 142 mS cm^–1^ at 80 °C) and the commercially available AEM FAA-3-50 at the same temperature (σ = 80 mS cm^–1^ at 80 °C), signifying its superiority in AEMWE applications. Table 3 lists the WU, SR, and σ for all of the PAP membranes prepared.

**Table 3:** WU, SR, and σ of *p*QPx-*m/p*TP Copolymers

	WU^a^/%		areal SR^b^/%		through-plane SR^c^/%		σ^d^/mS cm^–1^	
sample	25 °C	80 °C	25 °C	80 °C	25 °C	80 °C	25 °C	80 °C
M0	87	103	28	34	/	/	66^e^	142^e^
*p*QP25-*m*TP	28	80	30	104	24	44	64 ± 4	137 ± 4
*p*QP50-*m*TP	40	85	38	88	27	51	80 ± 3	197 ± 6
*p*QP25-*p*TP	37	67	21	68	18	40	80 ± 8	172 ± 2
*p*QP50-*p*TP	33	58	22	87	15	35	66 ± 1	145 ± 1

a Water uptake.

b Areal swelling ratio.

c Through-plane swelling ratios measured for one test sample.

d Conductivity values provided as an average of three samples along with the SD.

e Conductivity data from one test sample.

**Figure 7:**
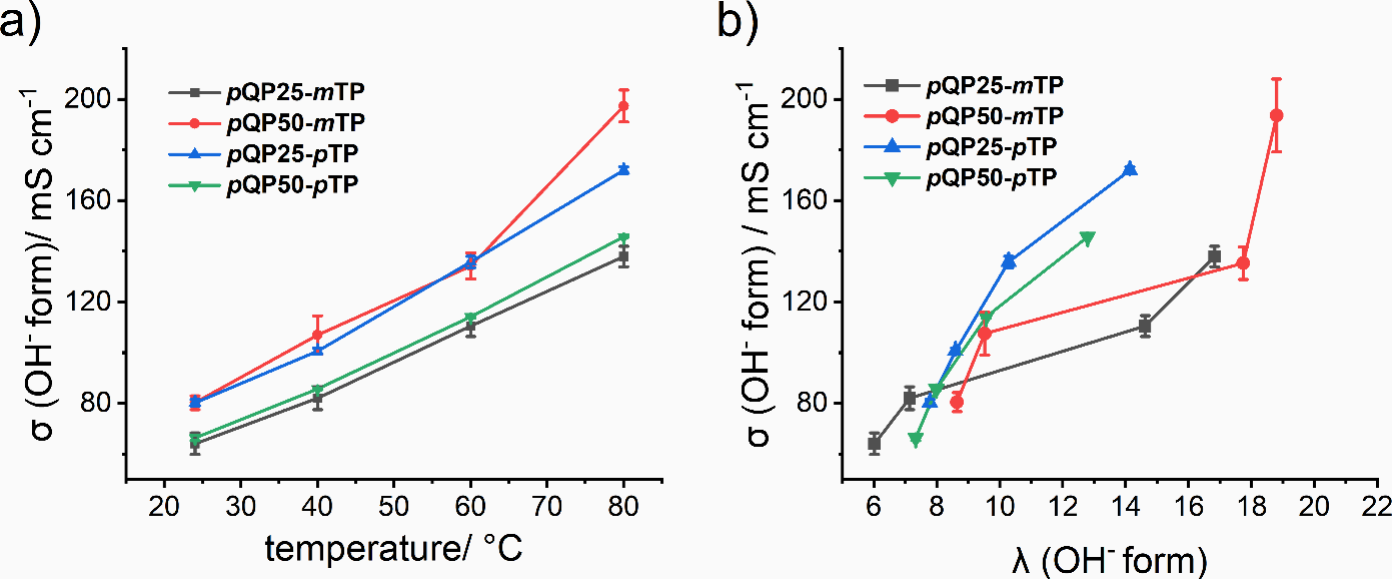
a) Hydroxide conductivity of *p*QPx-*m/p*TP as a function of temperature and b) hydration number.

### 3.7 Alkaline Stability

The piperidinium cations attached covalently to the copolymer backbones are key components for the hydroxide ion transport ability of the *p*QPx-*m/p*TP membranes. At the same time, they are susceptible to degradation via hydroxide attack in the otherwise stable phenyl-based backbone.^59^ The alkaline stability was evaluated by immersing the membranes in the OH^–^ form in different alkali solutions with different concentrations over a varied period of time. ^1^H NMR spectroscopy was used to evaluate the extent of degradation of the as-prepared *p*QPx-*m/p*TP membranes. Three mol% TFA was added to each NMR sample so as to shift the water signal beyond 10 ppm. In the first method, the membrane samples were tested under harsh conditions by immersing them in 5 M KOH solutions at 80 °C. They were removed after every 168 h (7 days), thoroughly washed with DI water, ion exchanged to chloride forms, dried under vacuum, and then subjected to ^1^H NMR analysis. Figure 8a shows the plot of ionic loss as a function of time for alkali treatment. Degradation is predominantly due to Hoffmann elimination reactions under high pH. The extent of degradation due to nucleophilic substitution gradually increases with time.^60^

**Figure 8:**
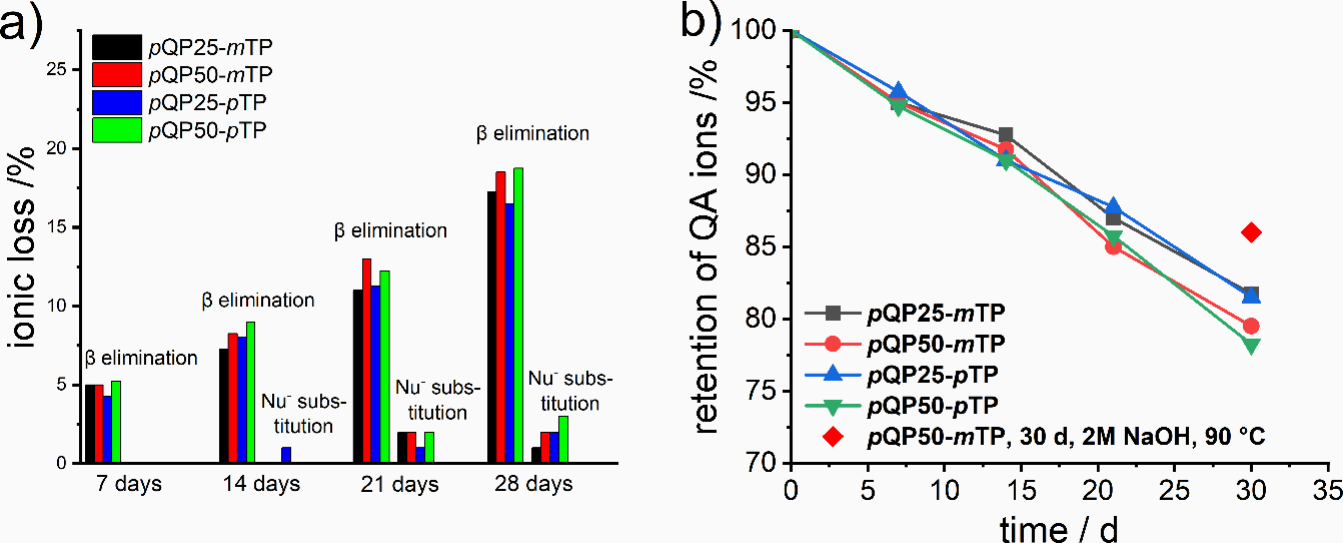
a) Plot of ionic loss versus the no. of days the *p*QPx-*m/p*TP membranes were treated in 5 M NaOH at 80 °C and b) percent retention of QA ions versus time of alkali treatment.

Figure 8b shows the remaining ionic content as obtained after calculating the degradation due to Hoffmann elimination, as well as nucleophilic substitution of the QA moiety at different durations. The four copolymers behave similar, with at least 80% of the ammonium groups remaining in all membranes even after 672 h under harsh alkaline conditions. Thus, the *p*QPx-*m/p*TP membranes prepared in this work show higher alkaline stability compared to similar ones reported in the literature.^35,47,61^ Additionally, to provide a better comparison of the alkaline stability for the *p*QPx-*m/p*TP membranes prepared in this work to that of the PAP membranes reported in the literature, we also carried out stability measurements at 2 M NaOH at 90 °C.^43,60,62^ The *p*QP50-*m*TP membrane (OH^–^ form) was immersed in 2 M NaOH for 30 days at 90 °C, and the stabilities were determined analogously. The *p*QP50-*m*TP membrane retained ∼86% (Figure 8b) of its original ionic content after 30 days, which is higher as compared to similar fluorine-free copolymers reported in the literature and measured, for which alkaline stabilities were determined under identical conditions.^47^ Figure 9 shows possible pathways for the degradation of QA ions under alkaline conditions based on signals showing up in the ^1^H NMR spectra of the *p*QP50-*m*TP membrane following alkaline degradation.

**Figure 9:**
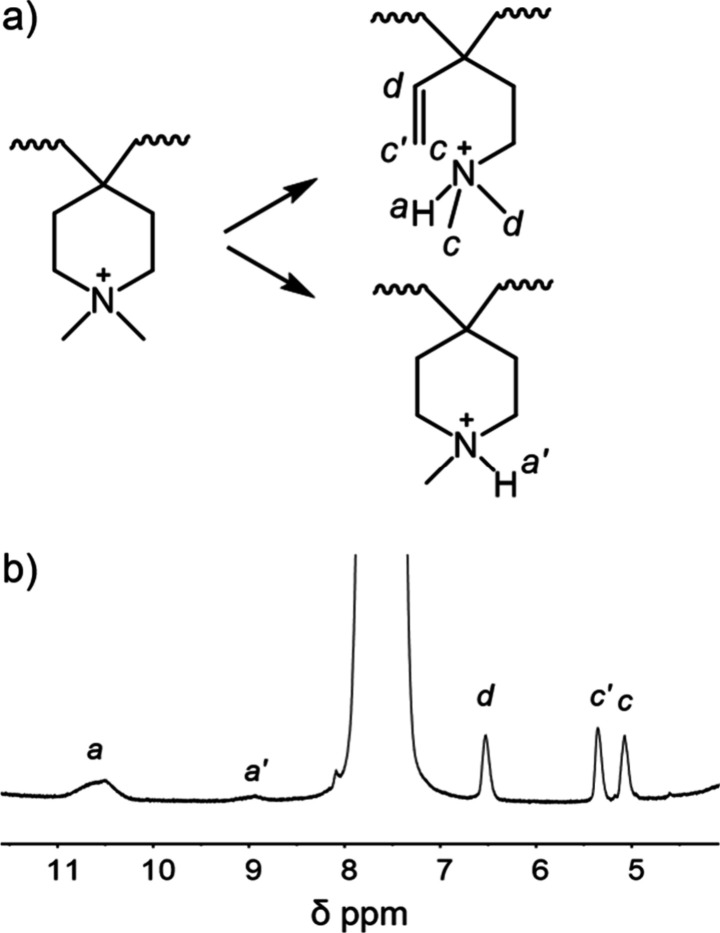
a) Possible pathways for degradation of QA ions under alkaline conditions based on b) ^1^H NMR spectra of *p*QP50-*m*TP after treatment in 2 M NaOH for 30 days at 90 °C measured in DMSO-*d*_6_.

## 4 Conclusions

The preparation and characterization of a set of four fluorine-free statistical poly­(arylene piperidinium) copolymers have been reported using the inexpensive monomers *para*-quaterphenyl (*p*QP), *meta*- (*m*TP) and *para*-terphenyl (*p*TP), and *N*-methyl-4-piperidone. Variation of the content of *p*QP and *m-* and *p*TP monomers resulted in high molar mass polymers that are still sufficiently soluble and formed membranes that featured attractive property profiles. Compared to copolymers without *p*QP and without the terphenyls, a significantly increased hydrophobicity and solubility, respectively, were achieved. Their tunable chemical structure and similar molar masses allowed for a comparative study and the optimization of key membrane properties, such as processability from solution, mechanical strength, ion exchange capacity, water uptake, hydroxide conductivity, and alkaline stability. Since terphenyl has a lower molar mass compared to quaterphenyl, the share of TP monomer allows us to fine-tune the hydrophilic/hydrophobic balance, IEC, and thus water uptake. SAXS patterns of hydrated membranes revealed pronounced ionomer peaks, supporting a phase separated morphology for poly­(arylene piperidium)­s containing *p*QP in their backbones. Using *m*TP instead of *p*TP as comonomer, strain at break, water uptake, and hydroxide conductivity significantly increased. Alkaline stabilities were superior compared to similar chemical structures under identical conditions. The copolymer membrane *p*QP50-*m*TP with a share of 50% *m*TP exhibited the most optimized property profile with a modulus and elongation at break of 318 MPa and 170% under submersed conditions, respectively, and a high hydroxide conductivity of 197 ± 6 mS cm^–1^ at 80 °C at a water uptake of 85% corresponding to a λ of 19. Overall, the presence of *m*TP renders the chain more flexible, which in turn leads to better packing but at the same time to a stronger temperature dependence of WU. These results demonstrate that fluorine-free polymer membranes made from commercial and simple monomers can be optimized to meet a variety of key requirements of AEM materials. They also show that subtle changes in comonomer composition lead to relatively strong changes in membrane characteristics and that fully understanding structure–function relationships remains a challenge. This work further highlights the potential of PAP polymers to bring forward the field of AEM water electrolysis by providing cost-efficient, fluorine-free, and high-performance membranes, thereby advancing the development of green hydrogen production technologies.

## Supplementary Material



## Notes

^1^H NMR spectra of prepared copolymers with TFA as counterion, chromatograms as obtained from SEC measurements, fitting parameters for Gaussian fits of scattering data, and Arrhenius plot of the *p*QPx-m/pTP samples (PDF)

## ○

Department of Electrification and Reliability, RISE Research Institutes of Sweden, 50462 Borås, Sweden
