# Supplementary material for: Kinking Matters: meta-Terphenyl Improves Hydroxide Conductivity of Mechanically Robust Fluorine-Free Poly(arylene piperidinium) Copolymers for Anion Exchange Membranes
Source: ACS Appl Mater Interfaces. 2025 Jul 10;17(29):41978–90. doi: 10.1021/acsami.5c08476 (PMC12291086; doi:10.1021/acsami.5c08476)
Supplement: Supplementary file 1 [file am5c08476_si_001.pdf]

Supporting Information

Kinking matters: *meta*-terphenyl improves hydroxide conductivity of mechanically robust fluorine-free poly(arylene piperidinium) copolymers for anion exchange membranes

Kajari Mazumder[a], Hannes Nederstedt[a,b], Richard Weber[a], Shuichi Haraguchi[c], Richard Neubert[d], Felix A. Plamper[d,e,f], Christian Müller[c], Michael Sommer*[a,g]

michael.sommer@chemie.tu-chemnitz.de

[a]Institut für Chemie, TU Chemnitz, Professur Polymerchemie, Straße der Nationen 62, 09111 Chemnitz, Germany

[b]Present address: Department of Electrification and Reliability, RISE Research Institutes of Sweden, 50462 Borås, Sweden

[c]Department of Chemistry and Chemical Engineering, Chalmers University of Technology, 41296 Göteborg, Sweden

[d]Institute of Physical Chemistry, TU Bergakademie Freiberg, Leipziger Straße 29, 09599 Freiberg, Germany

[e]Center for Efficient High Temperature Processes and Materials Conversion ZeHS, TU Bergakademie Freiberg, Winklerstr. 5, 09599 Freiberg, Germany

[f]Freiberg Center for Water Research ZeWaF, TU Bergakademie Freiberg, Winklerstr. 5, 09599 Freiberg, Germany

[g]Forschungszentrum MAIN, TU Chemnitz, Rosenbergstraße 6, 09126 Chemnitz, Germany

Contents

1. ^{1}H NMR spectra of prepared copolymers with TFA^{-} as counter ion…. Page S3
2. Chromatograms as obtained from SEC measurements………………. Page S5
3. Fitting parameters for Gaussian fits of scattering data……………… Page S7
4. Aging effects……………………………………………………. Page S8
5. Arrhenius plot of the *p*QPx-*m/p*TP samples…………………………. Page S10

1. ^{1}H NMR spectra of prepared copolymers with TFA^- as counter ion.

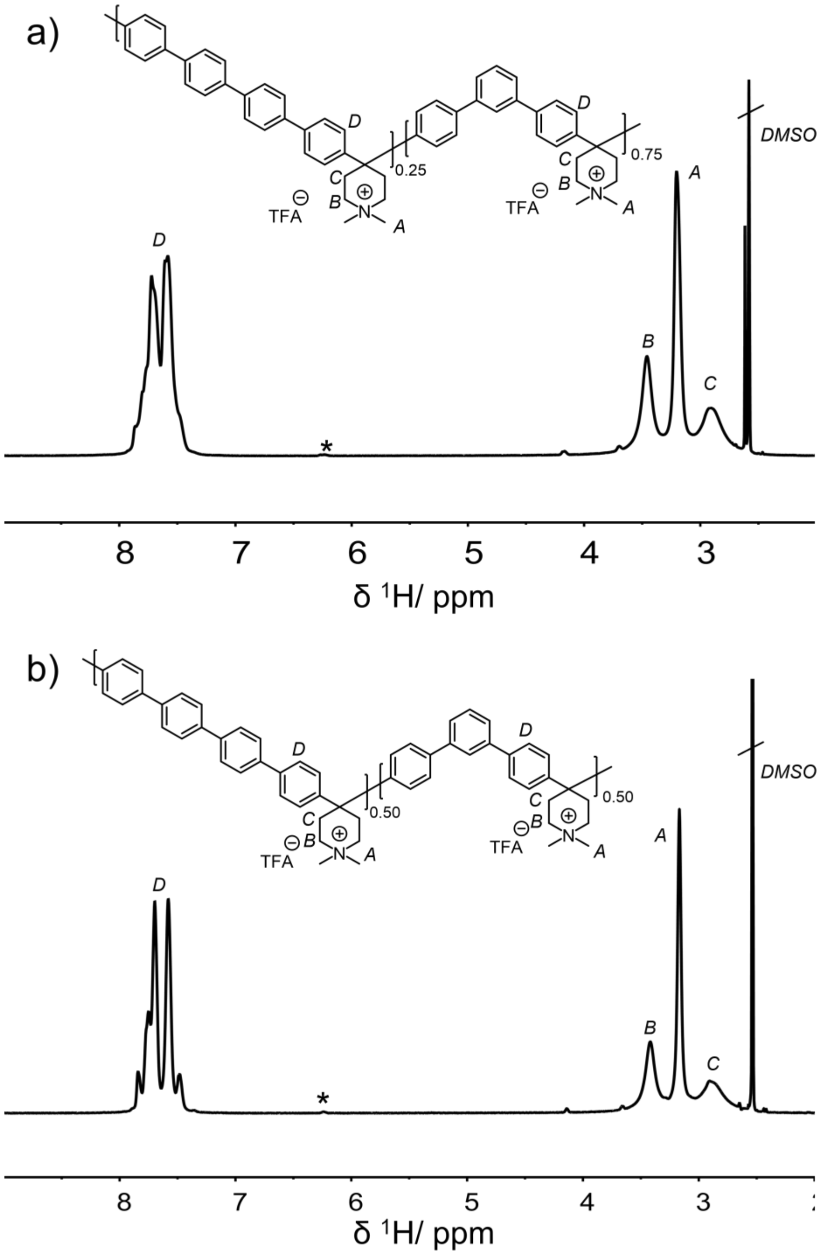

Figure S1. ^{1}H NMR spectra of a) *p*QP25-*m*TP and b) *p*QP50-*m*TP in DMSO-d_6 + TFA, * end group caused by elimination.

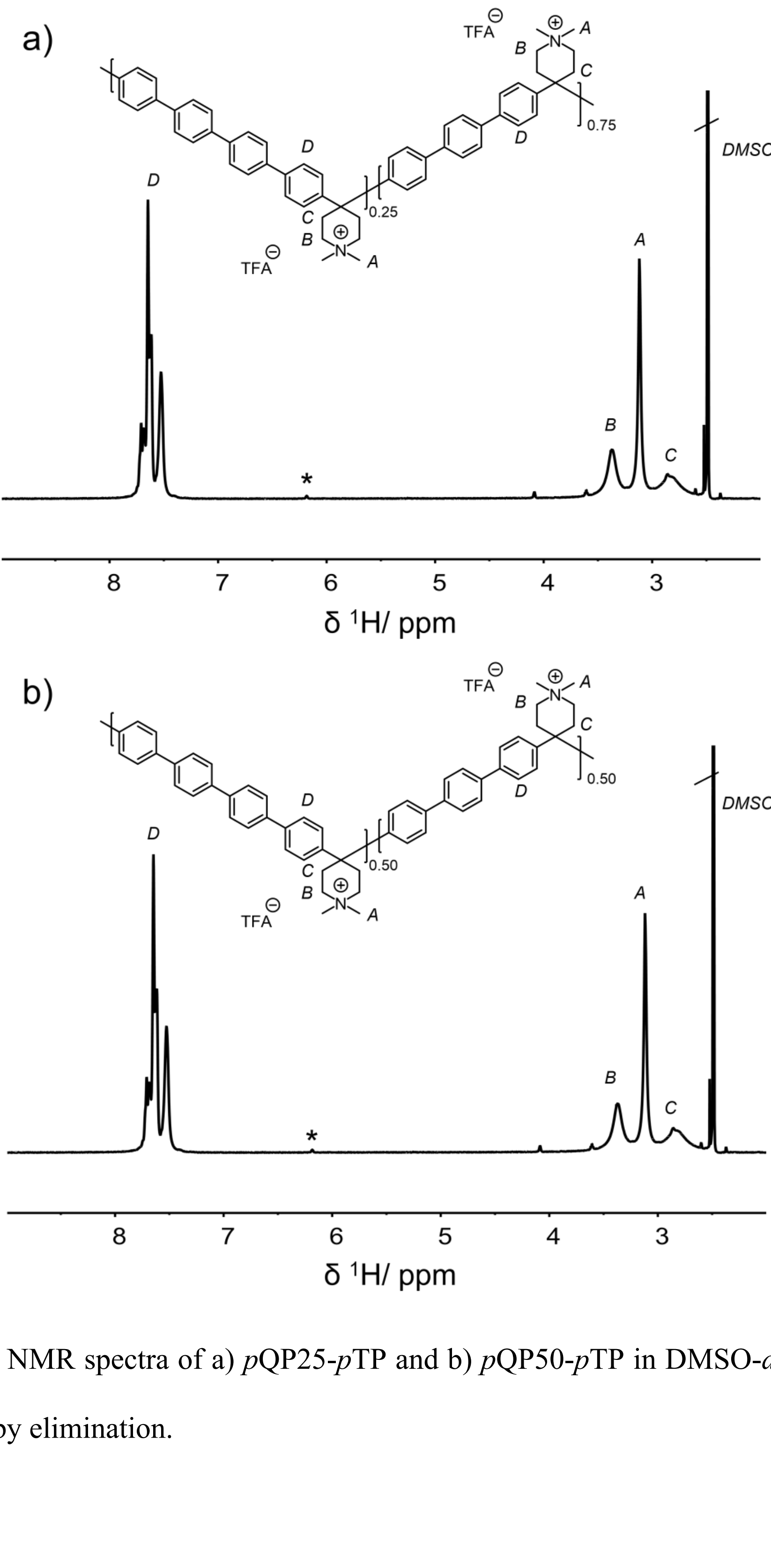

Figure S2. ^{1}H NMR spectra of a) *p*QP25-*p*TP and b) *p*QP50-*p*TP in DMSO-d_6 + TFA, ***** end group caused by elimination.

2. Chromatograms as obtained from SEC measurements.

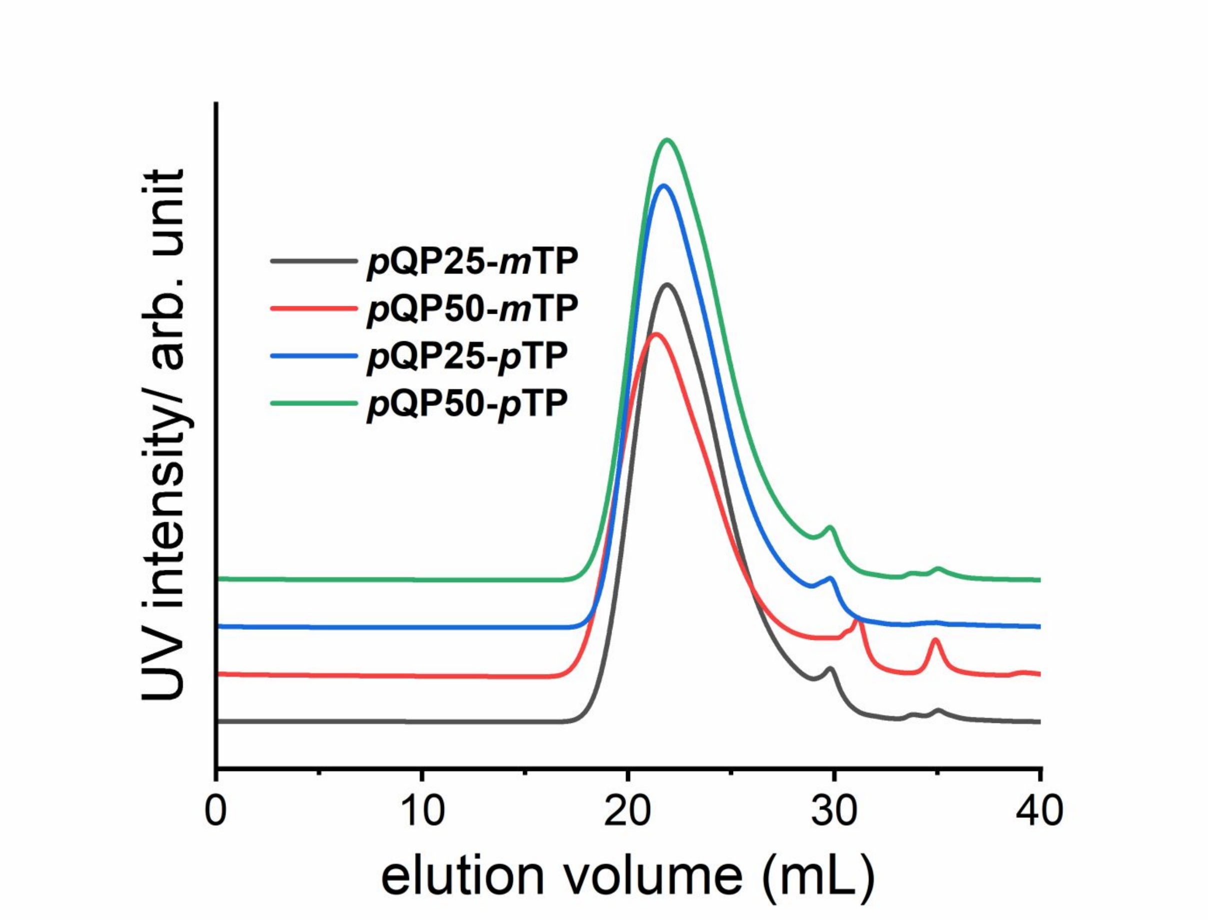

Figure S3. Chromatograms from SEC measurements of the quaternized polymers. Curves have been arbitrarily shifted along the y-axis for clarity.

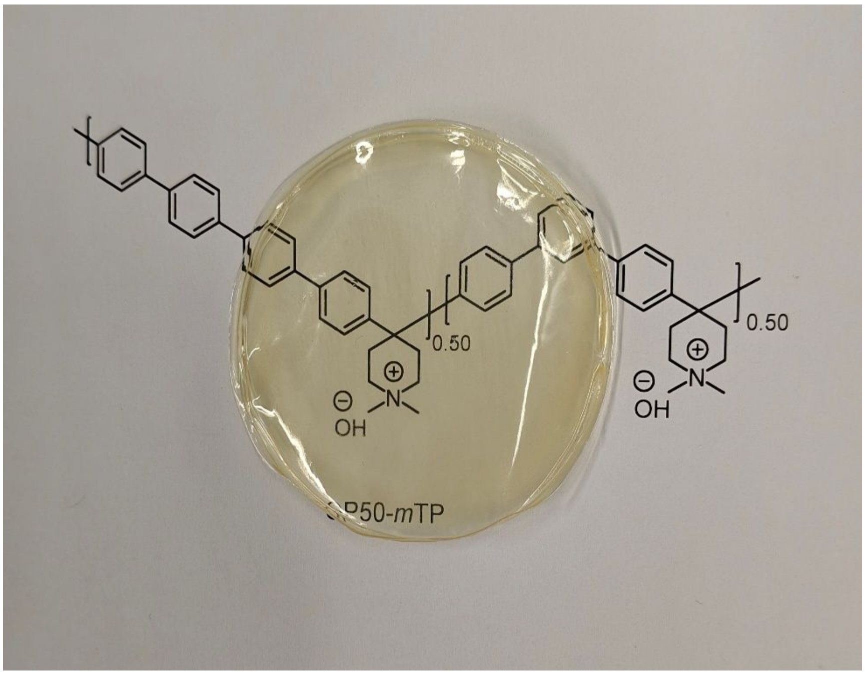

Figure S4. Photograph of membrane *p*QP50-*m*TP in chloride form with a thickness of ~150 µm.

3. Fitting parameters for Gaussian fits of scattering data.

Table S1: Fitting parameters for Gaussian fits of scattering data of Figure 6 (main article) | fitting range from 1.3 ≤ q [nm-1] ≤ 3.3 | fitting by SASview (Vers. 6.0.0.).

**χ^2**	**dataset**	**scale**	**scale (Err)**	**background in a.u.**	**background (Err) in a.u.**	**peak-pos. in nm^{-1}**	**peak_pos (Err) in nm^{-1}**	**σ**	**σ (Err)**
1.0226	*p*QP25-mTP	0.73297	0.0076476	0.19722	0.0075584	1.2083	0.010185	0.69013	0.014094
1.0863	*p*QP25-pTP	0.45335	0.0065867	0.49555	0.004711	1.4183	0.0065496	0.45581	0.0091197
1.0558	*p*QP50-mTP	0.64031	0.0068931	0.29266	0.0063011	1.4606	0.0057078	0.55258	0.0091534
1.9212	*p*QP50-pTP	0.80806	0.0052855	0.13737	0.0039491	1.2446	0.0055806	0.57999	0.0068039

The characteristic separation distance in the membranes was calculated using Bragg's equation:

$$d = \frac{2\pi}{q} \tag{S1}$$

where d is the average correlation length of the membrane (nm) and q is the scattering vector (nm^{-1}).

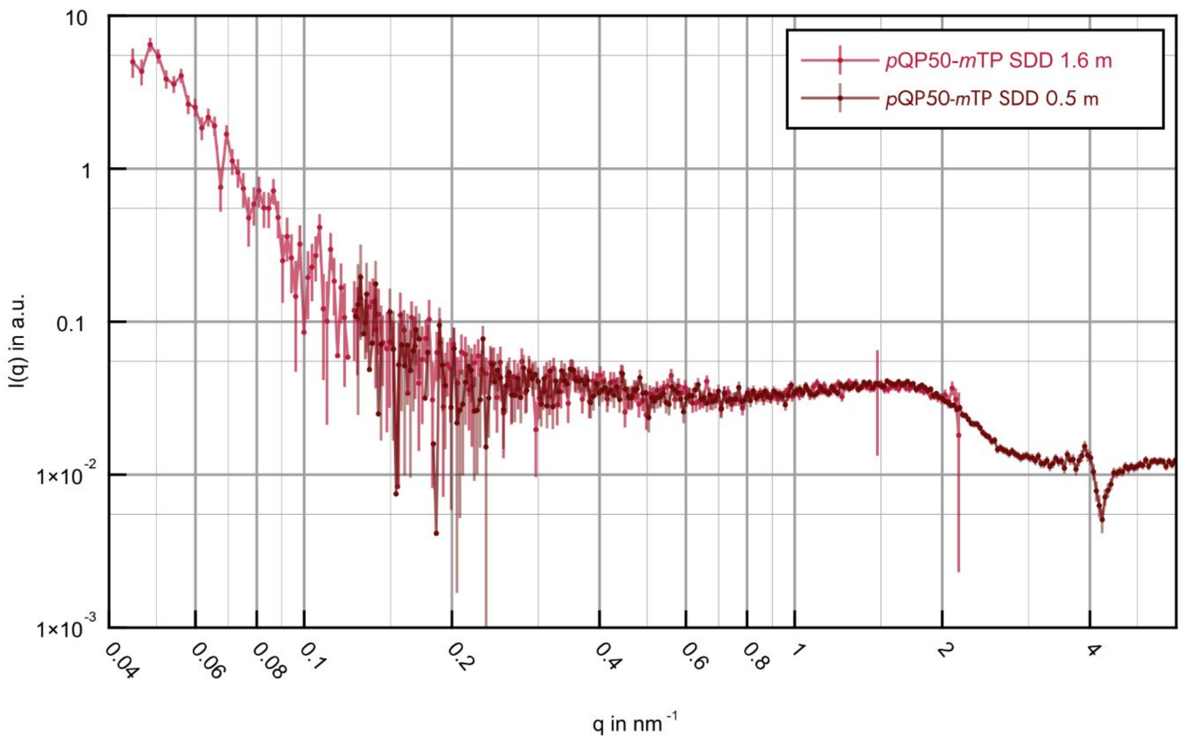

Figure S5. SAXS scans down to the limits of the device.

4. Aging effects.

Upon exposure to atmospheric carbonate, notable changes in the sample characteristics become apparent. In comparison to freshly produced samples stored under degassed water in sealed glass vessels and measured within 1 day after preparation, aged samples stored in normal atmosphere for 25 days were investigated. Extension of the q-range towards lower values reveals a secondary peak (Figure S6, $0.15 < q < 0.4$ nm^{-1}). For the *p*QP50-*p*TP sample, the corresponding domain size exceeds the measurement resolution, preventing clear peak identification. Further, the former better pronounced peak (main article, Figure 5) is now only visible as a shoulder. This might be connected to a difference in the equilibration time for the sample, since the measurement for the signal in Figure 5 (main article) was done 3 days after the preparation.

During the ageing process, a shift of the peak at small q values can be observed. While the secondary ionomer peak/shoulder remains largely unaffected, the peak regions begin to overlap due to the shift of the primary peak toward higher q-values. This shift corresponds to a reduction

in domain size, potentially associated with carbonate ion incorporation. This phenomenon is more pronounced in samples with 50 mol% *p*QP as compared to samples containing 25 mol% *p*QP, and in copolymers containing *p*TP relative to those containing *m*TP.

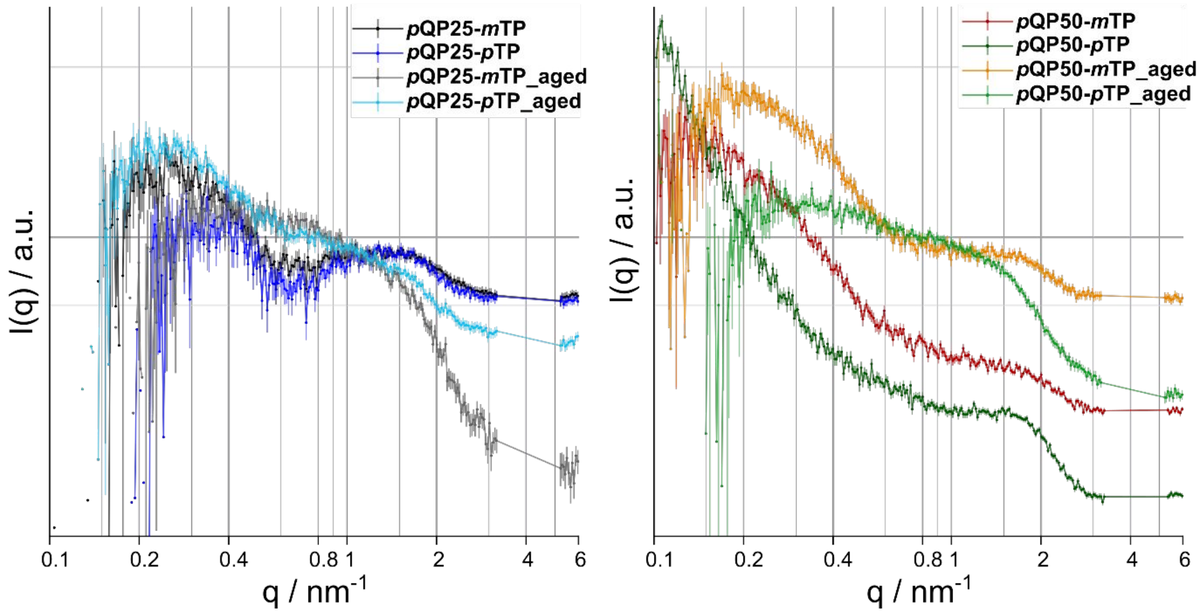

Figure S6. Scattering signal of fresh and aged membrane samples. Aged samples were stored in an open vessel with non-degassed water for 25 days before the measurement. The region from 3 to 5.5 nm^{-1} was masked (range of Kapton® peak).

5. Arrhenius plot of the *p*QPx-*m*/*p*TP samples.

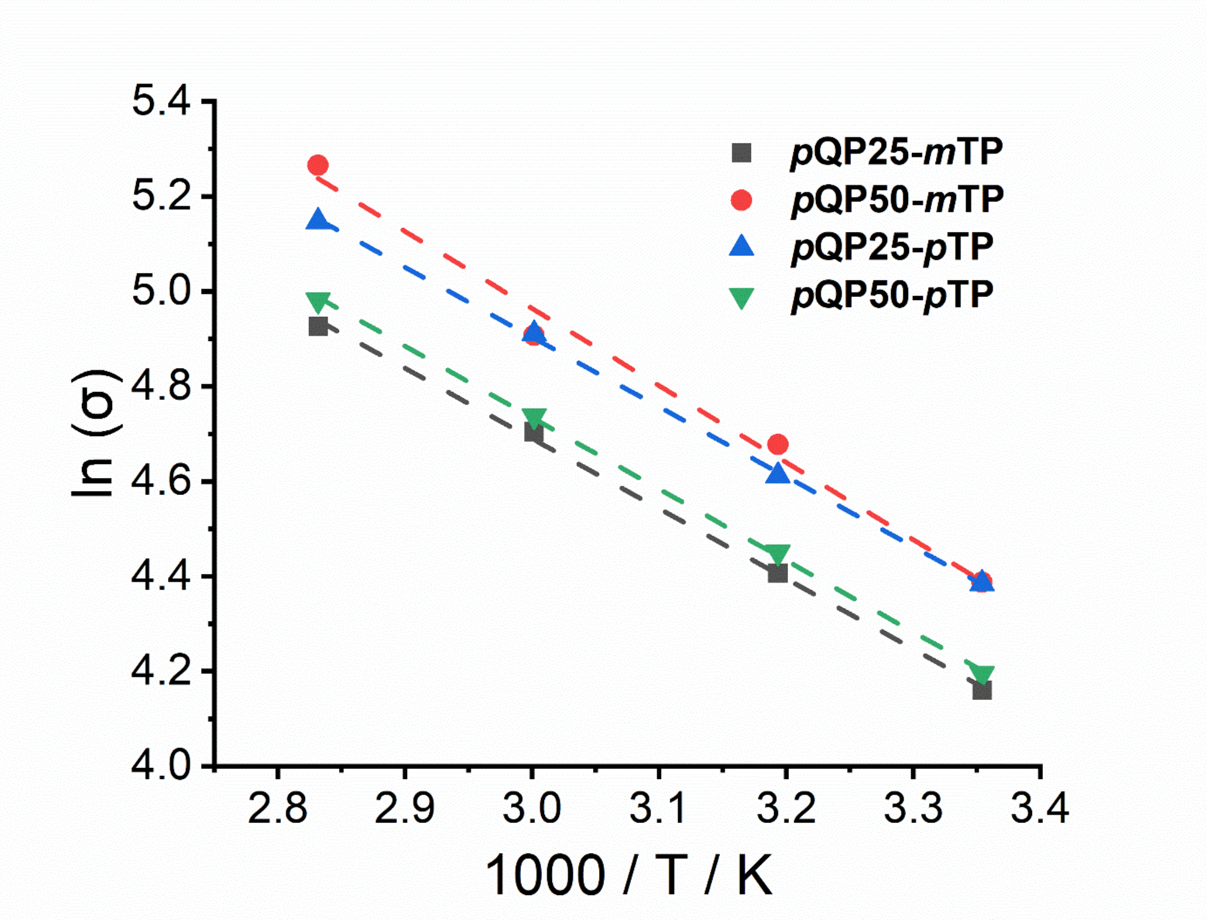

Figure S7. Arrhenius plot of the prepared *p*QPx-*m*/*p*TP samples.
